# Clear cell sarcoma of the parotid region

**DOI:** 10.5935/1808-8694.20120021

**Published:** 2015-11-20

**Authors:** Evandro Maccarini Manoel, Rafael Reiser, Fábio Brodskyn, Marcello Franco, Márcio Abrahão, Onivaldo Cervantes

**Affiliations:** a MD (Resident ENT Physician at EPM-UNIFESP); b MD, Head and Neck Surgeon (MD, Head and Neck Surgeon at EPM-UNIFESP); c MD, ENT, Head and Neck Surgeon (MD, ENT, Head and Neck Surgeon at EPM - UNIFESP); d MSc on Pathology at EPM-UNIFESP (Professor in the Department of Pathology at EPM-UNIFESP); e Adjunct Professor in the Department of Otorhinolaryngology and Head and Neck Surgery at EPM-UNIFESP (Vice-head of the Head and Neck Surgery Course at EPM-UNIFESP); f Adjunct Professor in the Department of Otorhinolaryngology and Head and Neck Surgery at EPM-UNIFESP (Head of the Head and Neck Surgery Course at EPM-UNIFESP and Chairman of the Brazilian Association of Head and Neck Surgery). Universidade Federal de São Paulo (EPM-UNIFESP)

**Keywords:** head and neck neoplasms, neoplasm recurrence, local, parotid region, sarcoma, clear cell

## INTRODUCTION

Clear cell sarcoma (CCS), also referred to as malignant melanoma of the soft parts, is a rare aggressive tumor that accounts for less than 1% of all soft tissue sarcomas^1^. It occurs typically as a deep lesion that arises in connection to tendons and aponeuroses, involving the skin only in advanced cases^2^. It is observed more frequently in adolescents and young adults of both genders, and preferentially affects the lower extremities^2^. It is rarely seen in the head or neck^3^.

## CASE PRESENTATION

A 43-year-old Caucasian female came to our service complaining of a lump that had been growing in her right parotid region for a year and four months. She had no other symptoms. The patient had well-managed systemic high blood pressure and asthma. Physical examination revealed a tumor in her right parotid region with a diameter of five centimeters. The tumor was hard, barely mobile, ulcerated, hyperemic, and painless to palpation.

**Figure 1:**
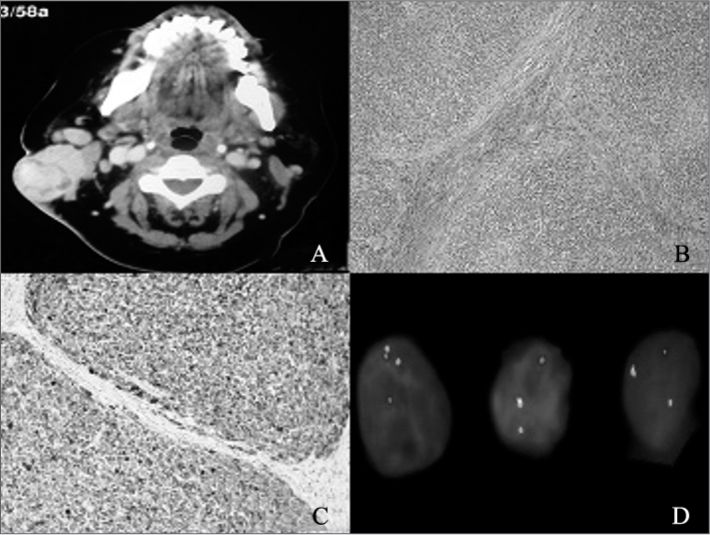
A: Head and neck CT scan showing a heterogeneous tumor with five centimeters in its greater diameter enhanced with contrast in the patient's right parotid region. B: optic microscope; H&E stained slide (magnification 100x). C: Immunohistochemistry assay with diffuse positive result for protein S-100 (magnification 200x). D: FISH test showing rearrangement in gene EWSR1 resulting from translocation t(12;22) (q13;12).

Fine-needle aspiration (FNA) revealed a basaloid neoplasm with low rates of cell proliferation. Head and neck CT scans showed a tumor located in the patient's right parotid region (Figure 1A).

The patient was referred to surgery and underwent a superficial parotidectomy with neck clearance on level II; the accessory nerve was removed as it had been involved by the tumor. Histopathology tests showed the tumor was an undifferentiated malignant neoplasm with a multilobular growth pattern and cells with little amounts of pale cytoplasm, vesicular nuclei, and occasionally prominent nucleoli (Figure 1B). Cell morphology initially indicated malignant melanoma metastasis, but immunohistochemistry revealed diffuse positive results only for protein S-100 (Figure 1C), and negative results for malignant melanoma markers HMB45, Melan-A, MART-1 and MITF. A FISH (fluorescence in-situ hybridization) test showed translocation t(12;22) (q13;q12) (Figure 1D) and changed the diagnosis to CCS.

Eight months after surgery the neck tumor recurred, and the patient was submitted to a radical neck clearance procedure and adjuvant radiotherapy (6600 cGy). The patient has been followed for six months since, and no relapsing tumors have been found.

## DISCUSSION

Clear cell sarcoma was first described in 1965^2^, and has been known as malignant melanoma of the soft parts because of the histological and immunohistochemical similarities it bears with melanomas^1^. However, molecular analysis revealed they are distinct tumors, as CCS presents translocation t(12;22)(q13;q12) that results in chimeric gene EWSR1/ATF1, which is not seen in melanomas^1^. This alteration is also seen in hyalinizing clear cell carcinomas of the salivary glands, angiomatoid fibrous histiocytomas, and in few cases of the recently described gastrointestinal subtype of CCS^4^. In this case, morphology and absence of melanocytic markers match the diagnosis of this variant^2^.

Only 1.2% of the approximately 500 reported cases^2^ of CCS involved the head or neck^5^. The parotid region was compromised in only three cases reported in the literature^3^^,^^5^^,^^6^. CCS generally evolves slowly and painlessly, and is diagnosed while measuring under five centimeters^2^. It presents high local recurrence and late metastasis rates^2^ and, contrary to most sarcomas, its metastases appear preferentially in regional lymph nodes. Five and ten-year survival rates are approximately 47% and 36% respectively^1^. Tumors larger than five centimeters and presence of tumor necrosis mean worse prognosis^6^. The better therapy appears to be broad excision of the tumor followed by adjuvant radiotherapy^2^. Given the limited number of reported cases, the role of the neck clearance procedure and systemic adjuvant therapy are still uncertain.

## CLOSING REMARKS

Even though they are rare, clear cell sarcomas may involve the head and the neck and are frequently mistaken for malignant melanomas.
